# Protective Effect of *Calculus Bovis Sativus* on Dextran Sulphate Sodium-Induced Ulcerative Colitis in Mice

**DOI:** 10.1155/2015/469506

**Published:** 2015-10-22

**Authors:** Xiping Li, Yanjiao Xu, Chengliang Zhang, Li Deng, Mujun Chang, Zaoqin Yu, Dong Liu

**Affiliations:** ^1^Department of Pharmacy, Tongji Hospital, Tongji Medical College, Huazhong University of Science and Technology, Wuhan 430030, China; ^2^Translational Medicine Center, Tongji Hospital, Tongji Medical College, Huazhong University of Science and Technology, Wuhan 430030, China

## Abstract

*Calculus Bovis Sativus* (CBS) is a commonly used traditional Chinese medicine, which has been reported to exhibit antispasmodic, fever-reducing, anti-inflammatory, and gallbladder-repairing effects. The present study aims to investigate the protective effect of CBS on dextran sulphate sodium- (DSS-) induced ulcerative colitis (UC) in mice. C57BL/6 male mice were exposed to 5% DSS in drinking water. CBS was given orally at 50 and 150 mg/kg once per day for 7 days. Body weight, disease activity index (DAI), colon length, colonic myeloperoxidase (MPO) activity, superoxide dismutase (SOD) activity, and malondialdehyde (MDA) and nitric oxide (NO) levels were measured. Administration of CBS significantly reserved these changes, decreased the MPO activity and MDA and NO level, and increased the SOD activity in the colon tissue. Histological observation suggested that CBS alleviated edema, mucosal damage, and inflammatory cells infiltration induced by DSS in the colon. Moreover, CBS significantly downregulated the mRNA expression of tumor necrosis factor-*α* (TNF-*α*), interleukin- (IL-) 1*β* and IL-6 in the colon tissue. Our data suggested that CBS exerted protective effect on DSS-induced UC partially through the antioxidant and anti-inflammatory activities.

## 1. Introduction

Ulcerative colitis (UC) is one of the two major forms of inflammatory bowel diseases (IBDs), which are chronic, idiopathic, relapsing, and inflammatory conditions that are immunologically mediated. Abdominal pain, diarrhea, bloody mucopurulent stool, and fistulization are the main clinical symptoms for UC [1, 2]. In Western countries such as North America and Europe, the incidence and prevalence in individuals with UC have increased rapidly in the past 50 years. World Health Organization has regarded UC as one of the most challenging health issues, and if the UC is not treated on time, the risk of colon cancer will be increased [3]. Although many studies suggest that genetic and environmental factors, infection, and immune system disorders are involved in the development of UC, its cause and underlying mechanisms remain unclear [2]. In addition, the kinds of drugs for the treatment of UC at present are not abundant and their effects are not satisfactory, so there is a demand for the development of novel and effective drugs [4]. Recent studies have revealed that oxidative stress and inflammatory pathway disorders may be involved in the pathogenesis of UC, and they become the new therapeutic targets for the treatment of UC [5, 6].

*Calculus bovis* (Niuhuang), as a commonly used Chinese medicinal material, was first recorded in* Shennong Bencao Jing* and has been used clinically in China for 2000 years due to sedative, antispasmodic, fever-reducing, anti-inflammatory, and gallbladder-repairing effects [7]. Since natural resource is scarce and expensive, people have endeavored for decades and eventually succeed to find its eligible substitute* Calculus Bovis Sativus* (CBS), also called* in vitro* cultured calculus bovis, with its properties and components included in the Pharmacopoeia of the People's Republic of China (2015 Edition) with a definite chemical profile [8, 9]. It has previously been reported that the therapeutic effect of CBS anti-inflammatory suppository (85.9%) was significantly better than that of salazosulfapyridine (75.0%) (*P* < 0.05) [10]. Our previous study also showed that CBS relieved *α*-naphthylisothiocyanate-induced liver injuries by enhancing reactive oxygen scavenging and by inhibiting lipid peroxidation [11]. Herein, we evaluated the effect of CBS on UC induced by dextran sulfate sodium (DSS) in mice and preliminarily explored the mechanism.

## 2. Materials and Methods

### 2.1. Chemicals and Reagents

Dextran sulfate sodium (DSS, MW 36000–50000) was purchased from MP Biomedicals, LLC (Aurora, Ohio, USA). CBS was provided by Wuhan Jianmin Dapeng Pharmaceuticals Co., Ltd., and suspended with pure water. The assay kits for determining the activities of superoxide dismutase (SOD) and myeloperoxidase (MPO) and the contents of malondialdehyde (MDA) and nitric oxide (NO) in colonic tissues were obtained from Jiancheng Institute of Biotechnology (Nanjing, China).

### 2.2. Experimental Animals

C57BL/6 male mice that were eight weeks old were provided by the Experimental Animal Center of Tongji Medical College, Huazhong University of Science and Technology. The animals were kept at room temperature (controlled at 25°C) with a light-dark cycle of 12 h each day. The experiments were carried out according to the National Institutes of Health Guide for the Care and Use of Laboratory Animals approved by the Animal Ethics Committee of Tongji Medical College, Huazhong University of Science and Technology.

### 2.3. Induction of DSS-Induced Experimental Colitis in Mice

Mice were divided into four groups (*n* = 8). Ulcerative colitis was induced by feeding animals 5% (w/v) DSS in drinking water. The animals were given free access to water containing DSS for seven days. Control animals received water without DSS. CBS (50, 150 mg/kg/day) was given orally once per day for seven days from the first day of induction. Mice in control group were given normal saline (containing 0.5% carboxymethylcellulose sodium, solvent of CBS) orally. The animals were weighed and monitored for the appearance of diarrhea and blood in the stool throughout the experimental period. The colon was excised, opened longitudinally, and rinsed with saline, and the length was measured.

### 2.4. Evaluation of the Disease Activity Index

The mice were checked daily for colitis based on body weight monitoring, gross rectal bleeding, and stool consistency. The overall disease severity was assessed by a clinical scoring system [12], and the disease activity index (DAI) score was calculated for each animal. Each score was provided as follows: diarrhea (0 = normal; 1 = slightly loose feces; 2 = loose feces; and 3 = watery diarrhea) and visible fecal blood (0 = normal, 1 = slightly bloody; 2 = bloody; and 3 = blood in whole colon). The DAI score ranged from 0 to 6 (total score).

### 2.5. Determination of MPO Activity

The colon (50 mg) was homogenized on ice in 1 mL lysis buffer (200 mM NaCl, 5 mM ethylenediamine tetraacetic acid (EDTA), 10 mM Tris, 10% glycerol, 1 mM phenylmethanesulfonyl fluoride (PMSF), 1 *μ*g/mL leupeptin, and 28 *μ*g/mL aprotinin; pH 7.4) by a homogenizer (Physcotron NS-51, Microtec, Chiba, Japan). The homogenate was centrifuged at 40,000 g for 2 min at 4°C, and the supernatant was stored at −80°C until assayed. The MPO activity in the colon was determined using the methods provided by the assay kit. The final MPO activity was represented as U/mg protein.

### 2.6. Determination of MDA and NO Level and SOD Activity in Colonic Tissue

The excised colons were homogenized in 0.1 M phosphate buffer (pH 7.4) using a Teflon homogenizer (Thomas Scientific, Swedesboro, NJ, USA) and the homogenates were centrifuged at 10,000 g for 15 min at 4°C. The supernatant was collected for the biochemical estimations. The levels of MDA and NO and the activity of SOD were all determined using the methods provided by the assay kits.

### 2.7. Histopathological Examination

Samples for histology were excised from the distal 6–8 cm of the colon, fixed in 10% buffered formalin, and embedded in paraffin blocks. Slices with 5 *μ*m sections were stained with hematoxylin and eosin (HE). Microscopic damage of the colonic mucosa was scored based on the degree of inflammation and the presence of edema and/or ulcerations (0 = normal; 1 = slight inflammation; 2 = moderate inflammation and/or edema; and 3 = heavy inflammation and/or ulcerations) [12].

### 2.8. Preparation of RNA from Tissue Samples

RNA was extracted from frozen colon using TRI reagent. The resulting solution was diluted 50-fold using Tris/EDTA buffer (TE buffer). RNA purity and concentration (*μ*g/mL) were calculated by absorbance measurement at 260 and 280 nm using a U-2800 spectrophotometer (Hitachi High Technologies, Tokyo, Japan).

### 2.9. Real-Time PCR

The cDNA was produced by using the SuperScript Preamplification System for first-standing cDNA synthesis. RT-PCR was performed on cDNA samples using the MiniOpticon RT-PCR System (Bio-Rad Laboratories, Hercules, CA, USA). Target gene expression was analyzed by real-time RT-PCR using the following primers: (forward 5′-3′, reverse 5′-3′): *β-actin*, GAGCGCAAGTACTCTGTGTG, CGGACTCATCGTACTCCTG;* IL-6*, CCCTGACAGACCCGGACTTA, GCCGAGACTGTTGTTCCATAAT;* TNF-a*, ATGGACACCAAACATTTCCTGC, CCAGTGGAGAGCCGATTCC; and* IL-1β*, CCTCTGCCAAGTCAGGTCTC, GAATGTGCCACGGTTTTCTT. To each well of a 96-well PCR plate, 25 *μ*L iQ SYBR Green Supermix, 3 *μ*L target gene forward primer (5 pmol/*μ*L), 3 *μ*L reverse primer (5 pmol/*μ*L), 4 *μ*L cDNA TE buffer solution, and 15 *μ*L RNase-free water were added. Real-time RT-PCR was conducted at a denaturation temperature of 95°C for 15 s, an annealing temperature of 56°C for 30 s, and an elongation temperature of 72°C for 30 s. The amplification fluorescence intensity was monitored using the MyiQ single-color real-time RT-PCR detection system (Bio-Rad Laboratories). The mRNA gene expression levels were normalized to *β*-actin gene expression.

### 2.10. Statistical Analysis

All the results were expressed as mean ± SD. Data analyses were performed using SigmaPlot 12.0 software (Systat Software Inc., USA). Statistical analysis was conducted using repeated measure ANOVA, one-way ANOVA, and Tukey's post hoc test. *P* < 0.05 was considered to indicate a statistically significant difference.

## 3. Results

### 3.1. Effect of CBS on Body Weight, DAI, and Colon Length of Mice

**Figure 1:**
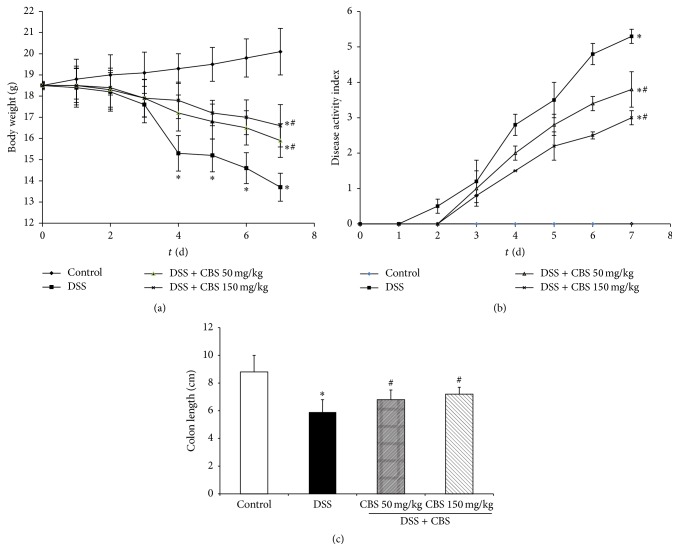
Effect of CBS on body weight and disease activity index of mice. (a) Body weight change; (b) disease activity index; and (c) colon length. Colitis was induced by administration with 5% dextran sulfate sodium (DSS) for 7 d. CBS (50 and 150 mg/kg) was administered daily for 7 d, respectively. Disease progression was monitored daily by observation of body weight change, disease activity index, and colon length change. Data are presented as mean ± SD of eight mice per group. ^*∗*^ *P* < 0.05 versus control, ^#^ *P* < 0.05 versus DSS alone.

Acute colitis manifested as diarrhea and bloody feces accompanied by a notable loss of body weight. In the DSS-treated group, body weight started to decrease from Day 4 of DSS administration and remained significantly decreased compared with normal mice until the 7th day (*P* < 0.05). The administration of CBS 50 mg/kg and 150 mg/kg significantly reversed the loss of body weight (*P* < 0.05) (Figure 1(a)). The DAI calculated by diarrhea and visible fecal blood was markedly higher in the DSS-treated group. CBS treatment suppressed DSS-induced colitis and ameliorated diarrhea and rectal bleeding (*P* < 0.05) (Figure 1(b)). In the DSS-treated group, the colon length was 5.9 ± 0.9 cm, significantly shorter than that of control mice (8.8 ± 1.2 cm) (*P* < 0.05). Treatment with different doses of CBS prevented colon shortening significantly (*P* < 0.05). The colon length was 6.8 ± 0.7 cm for CBS 50 mg/kg and 7.2 ± 0.5 cm for CBS 150 mg/kg (Figure 1(c)).

### 3.2. Effect of CBS on Colonic MPO Activity in DSS-Induced Ulcerative Colitis in Mice

**Figure 2:**
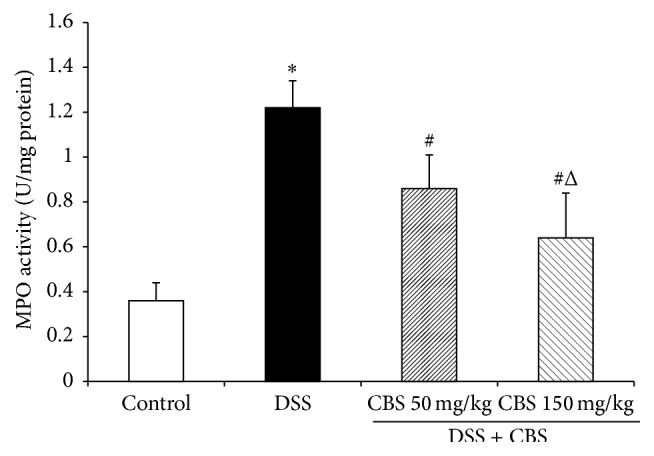
Effect of CBS on colonic myeloperoxidase (MPO) activity of mice. Colitis was induced by administration with 5% dextran sulfate sodium (DSS) for 7 d. CBS (50 and 150 mg/kg) was administered daily for 7 d, respectively. MPO activity was determined using an MPO detection kit. Data are presented as mean ± SD of eight mice per group. ^*∗*^ *P* < 0.05 versus control, ^#^ *P* < 0.05 versus DSS alone, and ^Δ^ *P* < 0.05 versus CBS 50 mg/kg.

The results showed that MPO activity in the DSS-treated group, an index of neutrophil infiltration, increased about 3-fold over that of control (Figure 2), showing a significantly difference. Following CBS (50 mg/kg and 150 mg/kg) treatment, MPO activity was reduced to 0.86 ± 0.15 and 0.64 ± 0.2 U/mg protein, respectively (*P* < 0.05).

### 3.3. Effect of CBS on MDA and NO Level and SOD Activity in Colonic Tissue of Colitis Mice

**Figure 3:**
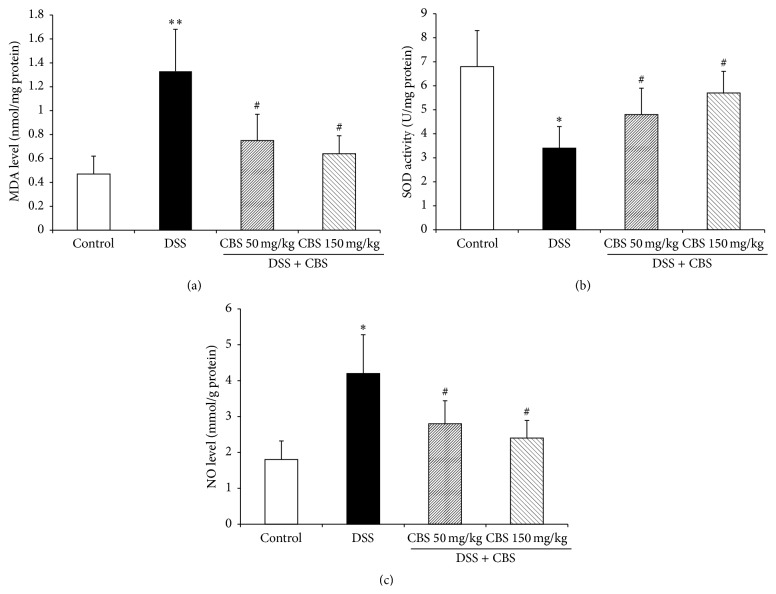
Effect of CBS on MDA level (a), NO level (b), and SOD activity (c) in colonic tissue of mice. Colitis was induced by administration with 5% dextran sulfate sodium (DSS) for 7 d. CBS (50 and 150 mg/kg) was administered daily for 7 d, respectively. Data are presented as mean ± SD of eight mice per group. ^*∗*^ *P* < 0.05 versus control, ^#^ *P* < 0.05 versus DSS alone, and ^*∗∗*^ *P* < 0.01 versus control.

As shown in Figure 3(a), DSS significantly increased the colonic MDA levels (1.33 ± 0.35 nmol/mg protein) compared with that of the normal control group (0.47 ± 0.15 nmol/mg protein) (*P* < 0.01). CBS treatment markedly reduced MDA levels to 0.75 ± 0.22 and 0.64 ± 0.18 nmol/mg protein at 50 mg/kg and 150 mg/kg, respectively (*P* < 0.05).

Induction of colitis significantly decreased the colonic SOD activity (3.4 ± 0.9 U/mg protein) compared with that of the control group (6.8 ± 1.5 U/mg protein) (*P* < 0.05). Treating mice with CBS (50 mg/kg or 150 mg/kg) for 7 days dose-dependently increased the SOD activity [4.8 ± 1.1 or 5.7 ± 0.9 U/mg protein] compared with that in the model group (*P* < 0.05) (Figure 3(b)).

In addition, DSS also elevated the colonic NO level compared with that of the control group (*P* < 0.05). The colonic NO level of DSS-colitis mice was significantly decreased following treatment with CBS (50 mg/kg or 150 mg/kg) for 7 days (*P* < 0.05) (Figure 3(c)).

### 3.4. Effect of CBS on Histopathological Changes in DSS-Induced Ulcerative Colitis in Mice

**Figure 4:**
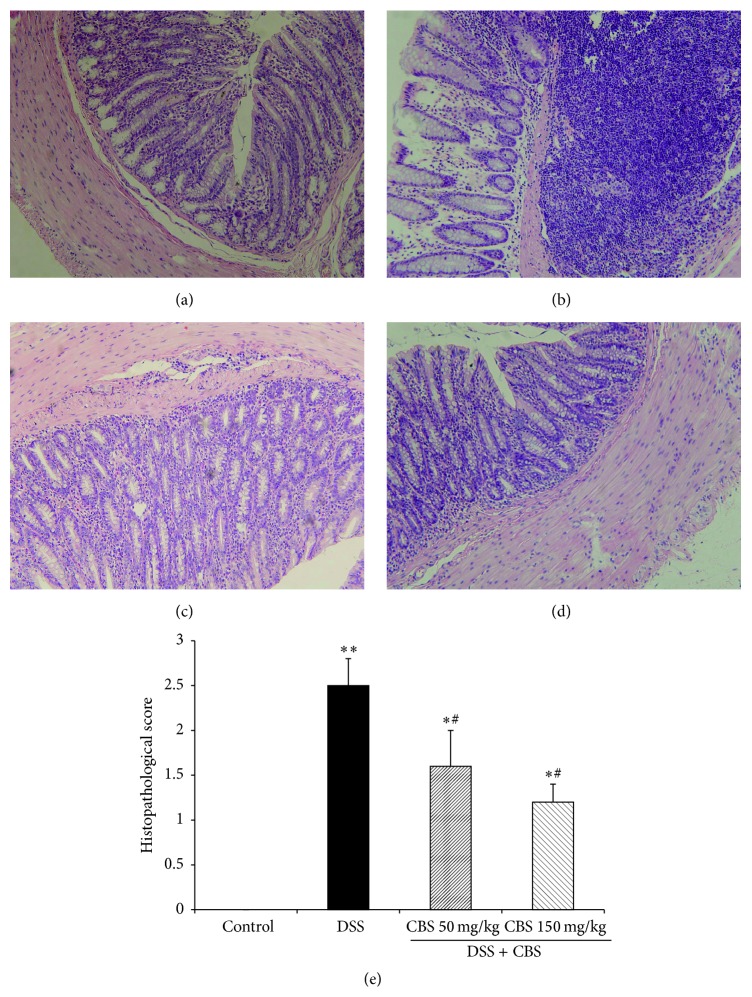
Effect of CBS on colonic histological observations in dextran sulfate sodium- (DSS-) treated mice. (a) Normal control group received water; (b) mice exposed to 5% DSS in vehicle for 7 days; (c and d) cotreatment with 50 and 150 mg/kg CBS, respectively, for 7 days together with DSS administration. Histopathological sections were stained by HE. Original magnification of 200x; and (e) histopathological scores. ^*∗*^ *P* < 0.05 versus control, ^#^ *P* < 0.05 versus DSS alone, and ^*∗∗*^ *P* < 0.01 versus control.

DSS-treated animals exhibited severe inflammation damage, as assessed from the colonic histopathological scores (range 0–3) (Figure 4(e)). The histological features of the colons of the control group mice were typical of a normal structure (Figure 4(a)) whereas the inflamed colon of mice with DSS-induced colitis showed evidence of mucosal edema, crypt distortion, thickening of the colon wall, and high level of inflammatory cells infiltration (Figure 4(b)). After treatment with CBS (50 and 150 mg/kg), the ulcer area significantly reduced, and edema and adhesion were alleviated. The inflammatory cells were less prone to infiltration and were localized in the mucosa (Figures 4(c) and 4(d)).

### 3.5. Effect of CBS on the mRNA Expression of Proinflammatory Cytokines in DSS-Induced Ulcerative Colitis in Mice

**Figure 5:**
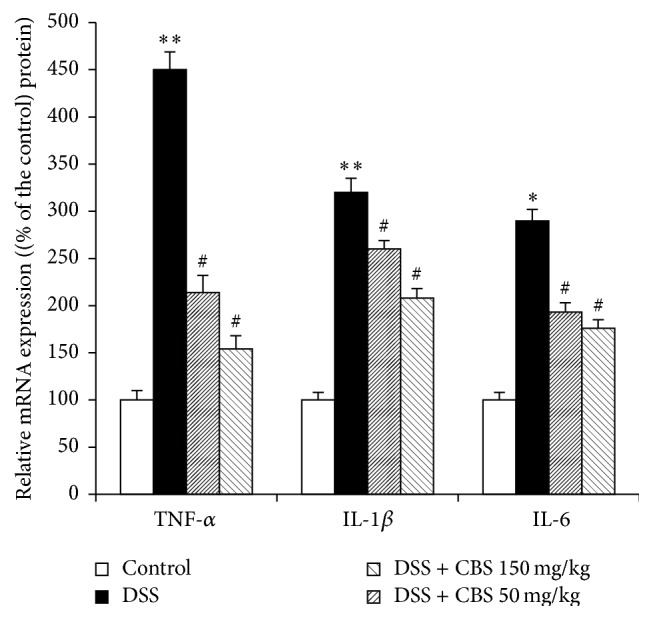
Effect of CBS on colonic mRNA levels of TNF-*α*, IL-1*β*, and IL-6 in dextran sulfate sodium- (DSS-) treated mice. Colitis was induced by administration with 5% DSS for 7 d. CBS (50 and 150 mg/kg) was administered daily for 7 d, respectively. Data are presented as mean ± SD of eight mice per group. ^*∗*^ *P* < 0.05 versus control, ^#^ *P* < 0.05 versus DSS alone, and ^*∗∗*^ *P* < 0.01 versus control.

To investigate the anti-inflammatory effects of CBS on DSS-induced colitis in mice, mRNA expression of TNF-*α*, IL-1*β*, and -6 in colonic tissue was analyzed by RT-PCR. As shown in Figure 5, colonic inflammation, induced by DSS, resulted in a markedly elevated expression of all proinflammatory cytokines. The administration of CBS effectively reduced the mRNA expression of TNF-*α*, IL-1*β*, and IL-6 in the colon tissue of mice with DSS-induced colitis.

## 4. Discussion

Establishment of UC by oral administration of DSS in the mouse is a widely used model for studies on this disease because this model resembles human UC and the resulting pathological conditions correspond very well to human. Following administration by gavage, most DSS is not absorbed and reaches the colon due to its large molecular weight and negative charge. The mechanism by which DSS induces intestinal inflammation is likely due to the damage to the epithelial monolayer lining in the colon, and the proinflammatory contents are reported to infiltrate underlying tissues [13].

In the present study, the UC model was successfully established by treating mice with 5% DSS for 7 days, and then the protective effect of CBS was investigated. After administration of CBS (50 and 150 mg/kg), the body weight loss, diarrhea, and colonic shortening by DSS were significantly reversed and improved (*P* < 0.05). In addition, DSS significantly increased MPO accumulation in the colon tissue. MPO is an index of colon mucosa neutrophil infiltration and its activity can positively reflect the number of neutrophil granulocytes [14]. It is also an important enzyme involved in free-radical generation, so increased MPO activity can aggravate tissue damage. In this study, treatment with CBS (50 and 150 mg/kg) significantly reduced the activity of MPO, decreased the lesion severity, reduced the extent of colon injury, and alleviated the infiltration of inflammatory cells induced by DSS. This confirms that CBS has a beneficial effect on UC.

Mice exposed to DSS alone exhibited greatly elevation of MDA level in the colon, suggesting induction of lipid peroxidation and excess production of reactive oxygen metabolites (ROMs) ensued [15]. Accumulative evidences showed that oxidative stress induced by excessively produced ROMs played an important role in the intestinal tissue damage of UC models [5, 16, 17]. CBS treatment significantly decreased MDA activity and NO content and increased the activity of SOD in DSS-induced UC. NO can promote the infiltration of neutrophils in the middle and distal colon, therefore leading to tissue damage. The antioxidant enzyme SOD, which converts cytotoxic superoxide radicals to H_2_O_2_, can defend the colon mucosa against oxidative damage caused by free radicals [18]. Taken together, those results demonstrated that CBS may protect against DSS-induced ulcerative colitis via regulation of oxidative stress biomarkers.

On the other hand, inflammatory responses play important roles in the pathogenesis of UC. The increased proinflammatory cytokines such as TNF-*α*, IL-1*β*, and IL-6 amplify the inflammatory cascade and result in intestinal tissue damage in UC induced by DSS [19]. Among those cytokines, the overexpression of TNF-*α* is vital in intestinal mucosal impairment [20]. Adalimumab, a TNF-*α* blocker, has been successfully used for the treatment of IBD patients in the clinic [21]. Another anti-TNF-*α* antibody Infliximab, a mouse monoclonal antibody, was also reported to exert effective therapeutic effect on UC in a clinical case [22]. In addition, IL-1*β* and IL-6 are two key mediators of the progression of UC. Inhibition the action of IL-1*β* and IL-6 can attenuate the severity of diarrhea and reduce infiltration of inflammatory cells into the intestinal tissue [23, 24]. In our study, the RT-PCR assay exhibited that the mRNA levels of TNF-*α*, IL-1*β*, and IL-6 were greatly reduced by CBS in DSS-induced colitis mice, suggesting that the protective effect of CBS against colonic injury is related to the regulation of TNF-*α*, IL-1*β*, and IL-6.

CBS, the dry gallstone of* Bos taurus domesticus* Gmelin, is complicated but unambiguous in terms of constituents [9]. In recent decades, it has been verified that the dominating therapeutic constituents therein are bile acids (unconjugated, taurine- or glycine-conjugated bile acids), taurine, and bilirubin [25]. Our previous studies have shown that CBS effectively enhanced reactive oxygen scavenging and inhibited lipid peroxidation, thus protecting rats from *α*-naphthylisothiocyanate- or ethinylestradiol-induced intrahepatic cholestasis [11]. The natural antioxidant bilirubin in CBS participated in activating antioxidative process [26]. Glycocholic acid (GCA), an unconjugated bile acid in CBS, is also reported to be useful for the treatment of gastrointestinal inflammations at oral or colonic administration [27]. Similarly, it is shown that deoxycholic acid (DCA) and lithocholic acid (LCA) could inhibit tumour necrosis factor-*α* production in macrophages and may have the potential to modulate immune responses in inflammatory bowel disease [28]. On the other hand, ursodeoxycholic acid (UDCA) was also reported to reduce mucosal damage and exert an anti-inflammatory activity in experimental colitis, and UDCA at the appropriate dose is a potentially useful drug to be incorporated in the treatment of IBD [29, 30].

Dietary taurine also exerts beneficial effects on both DSS- and trinitrobenzene sulphonic acid-induced experimental UC. Decreasing inflammatory reactions, oxidative stress and apoptosis may be involved in the mechanisms underlying the cytoprotective function of taurine in the intestinal epithelium [31, 32]. The taurine-conjugated bile acids in CBS such as tauroursodeoxycholic acid (TUDCA) and taurochenodeoxycholic acid (THDCA) were also proven to have protective effect on experimental colitis probably through its anti-inflammatory activities [33, 34]. In spite of this, the protective mechanism of CBS on DSS-induced UC is still unclear due to its complex components, and pharmacologically active component(s) targeting UC treatment is needed to be clarified next.

## 5. Conclusions

In conclusion, the findings in our study demonstrated that CBS can exert beneficial effects in DSS-induced ulcerative colitis model through its antioxidant effect and anti-inflammatory activity. And CBS may be regarded as a suitable candidate drug for the treatment of IBD. However, in regard to the complex composition of CBS, the underlying mechanism is still needed to be clarified in further study.
